# Identification of proteins associated with clinical and pathological features of proliferative diabetic retinopathy in vitreous and fibrovascular membranes

**DOI:** 10.1371/journal.pone.0187304

**Published:** 2017-11-02

**Authors:** Ingeborg Klaassen, Ewout W. de Vries, Ilse M. C. Vogels, Antoine H. C. van Kampen, Machteld I. Bosscha, David H. W. Steel, Cornelis J. F. Van Noorden, Sarit Y. Lesnik-Oberstein, Reinier O. Schlingemann

**Affiliations:** 1 Ocular Angiogenesis Group, Departments of Ophthalmology and Medical Biology, Academic Medical Center, University of Amsterdam, Amsterdam, The Netherlands; 2 Bioinformatics Laboratory, Clinical Epidemiology, Biostatistics and Bioinformatics (KEBB), Academic Medical Center, University of Amsterdam, Amsterdam, The Netherlands; 3 Department of Ophthalmology, VU University Medical Center, Amsterdam, The Netherlands; 4 Institute of Genetic Medicine, Newcastle University, Newcastle upon Tyne and Sunderland Eye Infirmary, Sunderland, United Kingdom; 5 The Harley Street Eye Clinic, London, United Kingdom; University of Florida, UNITED STATES

## Abstract

**Purpose:**

To identify the protein profiles in vitreous associated with retinal fibrosis, angiogenesis, and neurite formation in epiretinal fibrovascular membranes (FVMs) in patients with proliferative diabetic retinopathy (PDR).

**Methods:**

Vitreous samples of 5 non-diabetic control patients with vitreous debris and 7 patients with PDR membranes were screened for 507 preselected proteins using the semi-quantitative RayBio® L-series 507 antibody array. From this array, 60 proteins were selected for a custom quantitative antibody array (Raybiotech, Human Quantibody® array), analyzing 7 control patients, 8 PDR patients with FVMs, and 5 PDR patients without FVMs. Additionally, mRNA levels of proteins of interest were measured in 10 PDR membranes and 11 idiopathic membranes and in retinal tissues and cells to identify possible sources of protein production.

**Results:**

Of the 507 proteins screened, 21 were found to be significantly elevated in PDR patients, including neurogenic and angiogenic factors such as neuregulin 1 (NRG1), nerve growth factor receptor (NGFR), placental growth factor (PlGF) and platelet derived growth factor (PDGF). Angiopoietin-2 (Ang2) concentrations were strongly correlated to the degree of fibrosis and the presence of FVMs in patients with PDR. Protein correlation analysis showed PDGF to be extensively co-regulated with other proteins, including thrombospondin-1 and Ang2. mRNA levels of glial-derived and brain/derived neurotrophic factor (GDNF and BDNF) were elevated in PDR membranes. These results were validated in a second study of 52 vitreous samples of 32 PDR patients and 20 control patients.

**Conclusions:**

This exploratory study reveals protein networks that potentially contribute to neurite outgrowth, angiogenesis and fibrosis in the formation of fibrovascular membranes in PDR. We identified a possible role of Ang2 in fibrosis and the formation of FVMs, and of the neurotrophic factors NRG1, PDGF and GDNF in neurite growth that occurs in all FVMs in PDR.

## Introduction

Proliferative diabetic retinopathy (PDR) is a serious ocular complication of diabetes and is characterized by retinal neovascularization and microvascular leakage in response to chronic ischemia. Although anti-VEGF therapy alongside pan-retinal photocoagulation has been shown to reduce neovascularization and macular edema [1], response to anti-VEGF treatment is heterogeneous [2]. Additionally, there have been concerns that anti-VEGF treatment may temporarily increase fibrovascular membrane (FVM) formation and retinal traction [3–7]. Ultimately both retinal detachment and hemorrhages in PDR are the leading causes of permanent vision loss or blindness in adults of working age [8,9]. Therefore, it is imperative that the molecular pathways leading to FVM formation are better understood in order to identify novel therapeutic targets.

Recently, it has also become clear that PDR is characterized not only by fibrovascular but also by neuroglial pathology [10,11]. It was previously assumed that in PDR, the neurons of the retina are incapable of proliferation, and that the total neural cell volume remains either static, or is reduced due to apoptosis following diabetic damage [10]. Recently however, it has been shown that PDR-associated FVMs contain neurite extensions growing alongside Müller cells, a specialized type of retinal glia cell [12]. These neurites originate from rod photoreceptors and various populations of retinal ganglion cells [12–14]. New vessel growth is orchestrated by chemoattractant and trophic factors derived from neurons [15] and Müller cells [16], and Müller cells also serve as a scaffold for new vasculature [17]. Under these conditions, retinal glial cells, macrophages, monocytes, hyalocytes (resident cells in the vitreous), fibroblasts, pericytes and vascular endothelial cells will migrate and proliferate into the vitreous body, hereby forming FVMs [18,19]. The cause and pathological implications of early neurite recruitment in FVMs and their contribution to retinal angiogenesis and fibrosis are still unknown.

FVM formation occurs secondary to secretion of cytokines and growth factors by the retina in response to ischemia [4,20]. A number of cytokines and growth factors have been identified in measurable quantities in the vitreous of PDR patients, and their levels correlated strongly with PDR disease activity [21,22]. Among them, vascular endothelial growth factor (VEGF) remains the growth factor that is most-frequently studied [23], although various other growth factors such as transforming growth factor-ß (TGF- ß), hepatocyte growth factor (HGF), plasma kallikrein and platelet-derived growth factor (PDGF) have also been implicated in PDR [24–26]. Moreover, the balance between connective tissue growth factor (CTGF) and VEGF is correlated with the degree of retinal fibrosis [4] and the angio-fibrotic switch. The large number of growth factors and cytokines that may be involved in PDR pathogenesis necessitates the use of high throughput techniques to identify relevant proteins.

In the present study, we used a combination of semi-quantitative and quantitative antibody arrays to screen over 500 proteins in the vitreous of PDR patients and control patients operated for vitreous floaters. We included a large series of angiogenic and neurogenic growth factors to identify those that are involved in PDR pathogenesis and thus are potential therapeutic targets. In addition, we compared mRNA levels of the corresponding genes in FVMs of PDR patients and idiopathic epiretinal membranes (ERMs, macular puckers of non-diabetic patients) and various other retinal tissues to identify the possible sources of these proteins.

## Materials and methods

### Study population

For this study, 23 vitreous samples were used, including vitreous samples from PDR patients (n = 16) and from patients with vitreous floaters (n = 7). In addition, 21 FVMs were collected from patients with either PDR (n = 10) or non-diabetic patients with a macular pucker (n = 11) who were operated by pars plana vitrectomy. Clinical variables were assessed by trained ophthalmologists. The study was approved by the Medical Ethics Committees of the Academic Medical Center and the VU University Medical Center, Amsterdam, The Netherlands. The study was conducted according to the tenets of the Declaration of Helsinki and written consent was obtained from all patients.

Table 1 highlights clinical features of the 23 patients that were included in the study of proteins in vitreous. There was no statistically significant difference in the average age of control (58 years) and diabetic (52 years) patients (T-test, P = 0.27). Additionally, there was no difference in the gender ratio between both groups (Fischer’s exact test, P = 0.41). In total, 43% of participants in the study were male and 57% were female. The majority of the PDR patients received preoperative pan-retinal laser treatment (n = 14) and 9 out of 16 PDR patients received preoperative anti-VEGF therapy with bevacizumab (Avastin; Genentech, San Francisco, California, USA).

**Table 1:** Clinical characteristics of patients whose vitreous samples were used for protein analysis with antibody arrays.

Case	Status^1^	Ophthalmological Status	Age	Sex	Hemorrhage^2^	Degree of Fibrosis^3^	Degree of Neovascularisation^4^	Laser	Avastin	Array^5^
1	CON	Vitreal floaters	61	F	0	0	0	no	no	L+Q
2	CON	Vitreal floaters	59	F	0	0	0	no	no	L+Q
3	CON	Vitreal floaters	42	M	0	0	0	no	no	L+Q
4	CON	Vitreal floaters	51	F	0	0	0	no	no	Q
5	CON	Vitreal floaters	49	F	0	0	0	no	no	Q
6	CON	Vitreal floaters	68	M	0	0	0	no	no	L+Q
7	CON	Vitreal floaters	73	F	0	0	0	no	no	L+Q
8	DM2	PDR withoutFVM	54	F	1	0	0	yes	no	Q
9	DM2	PDR with FVM	44	M	3	3	2	yes	no	L+Q
10	DM2	PDR without FVM^6^	62	F	0	0	0	yes	yes	Q
11	DM2	PDR with FVM	56	M	2	2	2	yes	yes	L+Q
12	DM2	PDR with FVM	54	M	2	2	0	yes	no	Q
13	DM2	PDR with FVM	59	F	0	3	1	yes	yes	Q
14	DM2	PDR without FVM	63	M	1	0	2	yes	yes	Q
15	DM2	PDR without FVM	67	M	3	0	1	yes	no	Q
16	DM2	PDR with FVM	27	M	1	3	2	yes	yes	L
17	DM2	PDR with FVM	35	F	1	3	2	yes	yes	L
18	DM2 + INS	PDR with FVM	55	F	2	2	1	yes	no	Q
19	DM2 + INS	PDR with FVM	48	F	2	3	2	no	yes	L+Q
20	DM2 + INS	PDR with FVM	53	F	0	3	2	yes	no	L+Q
21	DM2 + INS	PDR without FVM	51	M	3	0	2	yes	no	Q
22	DM2 + INS	PDR with FVM	51	F	1	2	2	yes	yes	Q
23	DM2 + INS	PDR with FVM	57	M	0	3	2	no	yes	L

^1^ Diabetic status: CON, no diabetes; DM2, diabetes type 2; DM2 + INS, diabetes type 2 with insulin dependency.

^2^ Hemorrhage: 0, no hemorrhage; 1, mild hemorrhage; 2, moderate hemorrhage or heavy hemorrhage more than 2 months ago; 3, heavy hemorrhage within 2 months.

^3^ Degree of fibrosis: 0, no evidence of fibrosis; 1, few pre-retinal membranes; 2, membranes with limited extension into the vitreous; 3, abundant white membranes reaching into the vitreous body.

^4^ Degree of neovascularisation: 0, absent; 1, quiescent; 2, active.

^5^ Array used: L, L507 Array; Q, Quantibody Array.

^6^ Patient was operated for vitreomacular traction

Table 2 presents the clinical features of the 21 patients from whom membranes were harvested during pars plana vitrectomy. The patients where either non-diabetic and operated for the removal of a macula pucker (n = 11), or suffering from PDR and operated for the removal of FVMs (n = 10). PDR patients were on average younger (52 years) than control (71 year) patients (T-test, P = 0.002). No difference in gender ratio between both groups was found (Fischer’s exact test, P = 0.08). In total, 57% of the participants in the study were male and 43% were female. Eight out of 10 PDR patients had received pan-retinal laser treatment and another 8 out of 10 patients were treated with bevacizumab at 3 days prior to surgery.

**Table 2:** Clinical characteristics of patients whose fibrovascular membranes where used for mRNA analysis.

Case	Status^1^	Ophthalmological Status	Age	Sex	Hemorrhage^2^	Degree of Fibrosis^3^	Degree of Neovascularisation^4^	Laser	Avastin
1	CON	Pucker	84	F	0	1	0	no	no
2	CON	Pucker	66	F	0	1	0	no	no
3	CON	Pucker	72	M	0	1	0	no	no
4	CON	Pucker	79	M	0	1	0	no	no
5	CON	Pucker	62	F	0	1	0	no	no
6	CON	Pucker	73	F	0	1	0	no	no
7	CON	Pucker	70	M	0	1	0	no	no
8	CON	Pucker	66	F	0	1	0	no	no
9	CON	Pucker	80	F	0	1	0	no	no
10	CON	Pucker	63	F	0	1	0	no	no
11	CON	Pucker	69	M	0	1	0	no	no
12	DM1	PDR	41	M	3	2	2	yes	yes
13	DM1	PDR	59	M	2	1	2	yes	no
14	DM1	PDR	43	M	3	1	2	yes	yes
15	DM1	PDR	48	F	0	1	2	yes	yes
16	DM2	PDR	25	M	3	3	2	no	no
17	DM2 + INS	PDR	48	F	2	3	2	no	yes
18	DM2 + INS	PDR	79	M	3	1	2	yes	yes
19	DM2 + INS	PDR	39	M	1	1	2	yes	yes
20	DM2 + INS	PDR	72	M	3	1	2	yes	yes
21	DM2 + INS	PDR	61	M	1	2	2	yes	yes

^1^ Diabetic status: CON, no diabetes; DM2, diabetes type 2; DM2 + INS, diabetes type 2 with insulin dependency.

^2^ Hemorrhage: 0, no hemorrhage; 1, mild hemorrhage; 2, moderate hemorrhage or heavy hemorrhage more than 2 months ago; 3, heavy hemorrhage within 2 months.

^3^ Degree of fibrosis: 0, no evidence of fibrosis; 1, few pre-retinal membranes; 2, membranes with limited extension into the vitreous; 3, abundant white membranes reaching into the vitreous body.

^4^ Degree of neovascularisation: 0, absent; 1, quiescent; 2, active.

We repeated our analysis in an independent set of vitreous samples obtained by the University of Newcastle upon Tyne. A favorable ethical opinion for the collection of the samples was obtained from the National Health Service (NHS) research ethics committee (South East Coast—Surrey research ethics committee reference 12/LO/0130) and the collection carried out at Sunderland Eye Infirmary under the sponsorship of City Hospitals Sunderland NHS foundation trust. The study was conducted according to the tenets of the Declaration of Helsinki and written consent was obtained from all patients. Fifty two vitreous samples of PDR patients (n = 32) and of control patients without diabetes who had undergone vitrectomy for macular hole or non-inflammatory vitreous opacities (n = 20) were included. Eighteen of the PDR patients had fibrovascular membranes and 14 had no signs of fibrosis. PDR patients were on average younger (56 years) than control patients (66 years) (t-test, P = 0.01). In total, 27% of the 52 patients were male and 73% were female with no difference in gender ratio between both groups (Fischer’s exact test, P = 0.13). None of the PDR patients had received pan-retinal laser treatment or cataract surgery within the last 2 months, none had previous vitrectomy surgery and none had undergone previous treatments with steroid therapy in the last year. Patients with confounding retinovascular or other eye disease (e.g. retinal vein occlusion or glaucoma) and patients who had received anti-VEGF therapy within the last 3 months were excluded.

### Clinical measurements of PDR

The degree of fibrosis, activity of neovascularization, degree of hemorrhage, and presence and type of diabetes, were obtained from pre-operative ophthalmic and ultrasound examinations. Additionally, the patient files and per-operative observations, which were recorded using a standardized form, were used. Fibrosis was graded as 0 when there was no fibrosis, as 1 when there were a few pre-retinal membranes (limited as in macular pucker), as 2 when pre-retinal membranes were present with limited extension into the vitreous, and as 3 when abundant membranes reaching into the vitreous body were observed. Neovascularization was graded as 0 when absent, as 1 (quiescent) when only non-perfused vessels were present, and as 2 (active) when perfused pre-retinal capillaries were present [27]. Degree of hemorrhage was graded as 0 when all media were clear and all fundus details were visible, as 1 when media were slightly clouded but the fundus could still be examined, as 2 when the hemorrhage was moderate, or heavy and more than 2 months ago, and as 3 when the hemorrhage was heavy and less than two months ago.

### Control tissue and cells

Control retinas from donor eyes were provided anonymously by the Corneabank Beverwijk (http://www.eurotissuebank.nl/comeabank/), The Netherlands. In The Netherlands, the use of donor material is provided for by a law named "Wet op Orgaan Donatie (WOD)". Following this law, donors provide written informed consent for donation with an opt out for the use of left-over material for related scientific research. Specific requirements for the use of left-over material originating from corneal grafting for scientific research have been described in an additional document formulated by the Ministry of Health, Welfare, and Sport and the BIS foundation (Eurotransplant; Leiden, July 21, 1995; 6714.ht). The present study was performed in accordance with all requirements stated in the WOD and the relevant document and in accordance with these regulations approval of the local medical ethics committee was not required as the data were analyzed anonymously.

Human primary retinal pigment epithelial cells were also obtained from donor eyes, with a post mortem time of less than 15 hours, as described previously [28]. Human primary retinal endothelial cells and pericytes were obtained from donor eyes (up to 24 hours post mortem) as described for bovine retinas [29]. Endothelial cells were cultured in complete EBM-2 medium (Lonza, Breda, The Netherlands) with 5% human serum. For glial cells a human astrocytoma cell line, U-373 MG (Uppsala) (Sigma-Aldrich, Zwijndrecht, The Netherlands) was used. RNA from blood was obtained as described previously [30].

### Vitreous protein quantitation

Vitreous samples of 7 PDR patients with FVMs and of 5 control patients were measured using a biotin label-based human antibody array (Human Antibody L-series 507 Array; RayBiotech, Norcross, GA, USA) to screen for potential proteins of interest. The array was performed in accordance with the manufacturer’s instructions. In brief, vitreous samples were centrifuged for 15 min at 14,000 g at 4°C. Supernatant was collected and dialyzed with PBS overnight at 4°C. After determination of protein concentrations, the appropriate amount of biotin was added and samples were again dialyzed with PBS overnight at 4°C. Samples were then hybridized to the arrays overnight at 4°C with gentle shaking. Biotinylated proteins captured by the membrane-bound antibodies were detected by incubation with HRP-streptavidin and analysis was performed by an Agilent laser scanner (Agilent Technologies, Palo Alto, CA, USA). Spot intensities were quantified with ScanAlyze software (Michael Eisen, http://rana.lbl.gov/EisenSoftware.htm) and mean signal and median background values were applied in subsequent calculations.

Sixty proteins of interest (based on results of the L507 array and relevant literature) were selected for further quantitative analysis. Quantitation was achieved using a customizable array-based multiplex immunoassay (RayBiotech, Human Quantibody® array) in accordance with the manufacturer’s instructions. Standard curves for each protein were generated and the lower limit of detection (LOD) was calculated based on the average and standard deviation of four negative controls (average + 2x standard deviation). Proteins that were undetectable in more than half of the samples were deemed to be below the LOD.

### Correlation network construction

The correlation network was constructed in the statistical package ‘R’ (version 3.2.3) (https://www.R-project.org) using the igraph package (version 1.0.1) (igraph.org). First, Pearson correlations between Quantibody protein profiles over all samples were calculated for all pairs of proteins. Subsequently, the proteins were presented in a correlation network [27] in which the nodes represent proteins and the interconnecting lines represent the correlation between the proteins. We discarded all correlations that were lower than 0.7 and removed all unconnected proteins.

### RNA isolation and mRNA quantification

Fibrous tissue was removed during surgery, placed in 250 μL TRIzol reagent (Life Technologies, Carlsbad, CA, USA) and stored at -80°C until further processing. Membranes were then homogenized using a pestle and vortexed. RNA from ERMs was extracted and quantified using techniques described previously [28]. Total RNA yields were measured using a NanoDrop (ND1000 Spectrophotometer; NanoDrop Technologies, Wilmington, DE, USA) and RNA quality was assessed using an Experion™ Automated Electrophoresis System (Bio-Rad, Hercules, CA, USA). For all samples, RNA quality indicator (RQI) values ranged between 6.5 and 8.5.

A 1-μg aliquot of total RNA was treated with DNAse I (Amplification Grade; Invitrogen) and reverse transcribed into first strand cDNA using the Maxima® First Strand cDNA Synthesis Kit (Thermo Scientific, Roskilde, Denmark). Real-time quantitative PCR was performed on 20x diluted cDNA samples using a CFX96 system (Bio-Rad, Hercules, CA) as described previously [31].

Primer details are listed in S2 Table. The specificity of the primers was confirmed by NCBI BLAST. The presence of a single PCR product was verified by both the presence of a single melting temperature peak and detection of a single band of the expected size on 3% agarose gel. Non-template controls were included to verify the method and the specificity of the primers. Normalization of data was performed with global mean normalization [32].

### Statistical analysis

Values of vitreous proteins are reported as a mean (pg/ml) ± standard deviation. Univariable analysis with two-tailed T-tests assuming unequal variance were performed to identify individual proteins significantly associated with PDR. For all statistical analyses a P value < 0.05 was considered to indicate statistically significant differences.

## Results

### Detection of vitreous proteins

The L507 array allowed us to screen the vitreous for presence of 507 proteins in PDR patients with FVMs relative to control patients with floaters. 453 proteins were shown to have a higher and 54 proteins had a lower concentrations in vitreous of PDR eyes. Only 55 of the proteins were significantly higher in concentration in PDR patients, whereas none of the proteins were significantly lower in concentration in PDR patients (two-tailed Welch’s t-test). Fifty three proteins had concentrations in the vitreous of PDR patients more than 2-fold higher than controls, while 25 proteins had a 10-fold higher concentration (S1 Table).

### Quantitation of vitreous proteins

On the basis of the results of the L507 array and relevant literature, we designed a custom quantibody array containing antibodies for the detection of 60 proteins in 20 samples (13 PDR and 7 controls). Although more expensive, the advantage of the quantibody array is that actual protein concentrations rather than relative yields of protein can be measured. Of the 60 proteins screened, 20 had concentrations below the LOD (defined as >50% of samples below detection limit) and were excluded from further analysis (AR, b-NGF, BDNF, COCO, E-Selectin, GDNF, ICAM3, IGF1R, IGF2, Insulin, Insulin R, NT3, NT4, PDGF-Ra, PDGF-Rb, Prolactin, TARC, TGFβ1, TPO and XEDAR). For the other 40 proteins, occasional samples that were below the LOD were approximated as the LOD/√2 [33]. Four proteins showed concentrations above the highest standards (Adiponectin, IGFBP6, NrCAM and TIMP-1).

The results of the quantibody array and the lower limits of detection are presented in Table 3. Twenty-one proteins were significantly upregulated in PDR patients (Welch’s T-test) and are highlighted in bold. In addition, 14 of the proteins exhibited a larger than 3-fold rise in concentration and are highlighted in bold. In a second independent study group of PDR patients (n = 32) and controls (n = 20) from the University of Newcastle upon Tyne, we measured protein levels of 5 of these proteins (Ang2, NRG1β1, PDGF-AA, PlGF and VEGF). The fold changes between PDR patients and controls were comparable with those of the first study group and the differences were statistically significant, except for NRG1β1 (S3 Table).

**Table 3:** Protein concentrations in vitreous of control and PDR patients. Protein concentrations were determined by Quantibody arrays in patients with vitreal floaters (CON) and patients with proliferative diabetic retinopathy (PDR) that underwent vitreoretinal surgery.

		CON				PDR					
	LOD	mean	SD	min	max	mean	SD	min	max	Fold Change	P-value
Adiponectin*	5.5	9680.0	4869.9	2116.3	17181.5	38271.7	21622.7	6359.8	71881.8	3.95	<0.001
ANG-1	5.4	95.1	28.0	57.0	137.8	259.6	240.4	69.4	863.9	2.73	0.031
ANG-2	5.1	93.5	52.9	28.9	161.0	1802.4	2979.4	117.8	11369.4	19.28	0.061
AR	8.2	BDL				BDL					
BDNF	1.0	BDL				BDL					
bFGF	32.5	117.0	236.8	23.0	653.7	45.2	19.6	23.0	80.0	0.39	0.454
BMP-2	1.7	59.2	11.5	44.6	73.4	95.9	51.5	27.7	202.6	1.62	0.028
BMP-5	148.6	240.7	111.3	105.1	396.9	281.8	106.4	105.1	513.1	1.17	0.439
b-NGF	2.9	BDL				BDL					
COCO	418.2	BDL				BDL					
DcR3	242.7	202.1	52.1	171.6	282.1	656.5	655.2	171.6	2487.7	3.25	0.028
ErbB3	13.0	154.5	24.5	126.5	186.6	179.4	98.2	9.2	403.9	1.16	0.401
E-Selectin	50.2	BDL				BDL					
Galectin-3	32.5	162.9	284.2	23.0	798.7	353.1	497.5	23.0	1579.9	2.17	0.291
GDF-15	0.8	370.7	273.1	48.1	880.7	975.7	425.8	172.8	1645.4	2.63	0.001
GDNF	3.0	BDL				BDL					
GH	29.0	34.1	20.5	20.5	75.2	77.5	53.0	20.5	177.8	2.28	0.018
HGF	4.3	2500.6	1062.3	1618.6	4418.0	7967.1	2151.6	2752.1	10711.5	3.19	<0.001
ICAM-1	76.4	696.0	1031.4	54.0	2707.0	3762.4	3531.0	696.9	13167.3	5.41	0.011
ICAM-3	24.7	BDL				BDL					
IGFBP-1	4.3	222.5	242.9	38.6	726.1	2210.9	2132.1	207.7	7383.2	9.94	0.006
IGFBP-2	41.5	16751.2	7003.4	7562.7	30992.2	24718.9	9332.7	13573.6	42543.5	1.48	0.047
IGFBP-3	114.1	1043.7	704.0	319.4	2053.4	19596.4	11034.1	2595.4	37544.3	18.78	<0.001
IGFBP-4	539.2	3463.8	1293.0	2009.4	5910.7	3471.7	1488.8	1156.9	6029.0	1.00	0.990
IGFBP-5	115.4	234.3	125.6	81.6	393.4	389.6	366.1	81.6	1570.1	1.66	0.185
IGFBP-6*	68.3	27483.8	8803.2	22349.7	47174.6	33899.1	10489.7	21769.8	55469.0	1.23	0.168
IGF-I	27.0	51.8	41.1	19.1	129.4	62.3	40.2	19.1	151.5	1.20	0.594
IGF-I R	48.6	BDL				BDL					
IGF-II	41.4	BDL				BDL					
IGF-II R	18.4	199.2	47.5	148.0	266.4	208.7	190.9	27.2	770.8	1.05	0.867
Insulin	20.1	BDL				BDL					
Insulin R	3,115.9	BDL				BDL					
NCAM-1	104.1	17561.3	8210.6	5251.9	28434.0	17835.7	9907.3	6488.9	37545.7	1.02	0.948
NGF R	10.3	41.3	27.4	7.3	78.5	74.7	21.7	40.2	105.6	1.81	0.019
Notch-1	2.1	21.9	16.4	12.6	58.3	17.4	18.2	1.5	62.7	0.80	0.590
NOV	5.8	1836.0	458.4	1202.5	2740.6	3206.6	942.9	1627.7	5509.0	1.75	<0.001
NrCAM*	5.0	4872.9	1914.6	2185.5	7215.7	6082.5	3285.2	715.4	12610.8	1.25	0.313
NRG1-b1	10.8	11.9	4.8	7.6	19.9	36.9	27.5	7.6	69.7	3.10	0.007
NT-3	12.1	BDL				BDL					
NT-4	5.3	BDL				BDL					
PDGF Ra	1,170.8	BDL				BDL					
PDGF Rb	10.8	BDL				BDL					
PDGF-AA	2.0	99.1	86.8	9.6	263.3	602.2	556.6	91.8	1827.7	6.08	0.007
PDGF-AB	5.0	6.6	1.6	3.5	8.3	16.9	16.2	5.5	67.5	2.54	0.042
PDGF-BB	0.4	0.7	0.5	0.3	1.3	2.9	2.7	0.6	9.3	4.03	0.015
PIGF*	2.5	1.8	0.0	1.8	1.8	102.8	134.5	1.8	467.4	57.30	0.019
Prolactin	151.7	BDL				BDL					
TARC	1.7	BDL				BDL					
TGFb1	151.4	BDL				BDL					
TGF-b2	7.1	30.3	14.1	13.0	43.9	48.9	56.7	5.0	184.7	1.61	0.281
Thrombospondin-1	215.0	980.3	1650.2	152.0	4645.7	10116.4	10082.6	499.9	34455.3	10.32	0.007
TIMP-1*	13.4	60145.6	19198.7	41110.6	88938.0	61745.9	9704.1	47592.0	82709.5	1.03	0.842
TIMP-4	6.5	395.0	333.7	142.0	1054.3	611.8	282.4	196.5	1069.2	1.55	0.173
TPO	139.9	BDL				BDL					
Ubiquitin+1	136.8	261.9	197.4	96.7	578.9	897.7	390.9	271.1	1465.3	3.43	<0.001
VCAM-1	794.5	7383.1	7964.7	561.8	23677.8	8143.9	5254.2	2198.5	22644.2	1.10	0.825
VEGF	10.7	23.5	42.2	7.6	119.2	1208.1	1528.2	7.6	5038.6	51.40	0.016
WIF-1	19.5	11133.9	2970.5	8184.8	15679.3	9207.2	3063.2	4362.8	13964.5	0.83	0.195
WISP-1	68.6	176.1	98.6	48.5	293.8	191.5	121.9	48.5	499.5	1.09	0.763
XEDAR	3.3	BDL				BDL					

Data are presented in pg/mL ± SD with min and max values. BDL, below limit of detection (LOD). Unpaired t-test with Welch's correction was used to assess statistical differences between PDR and control patients. Fold changes higher than 3-fold and significant differences (P < 0.05) are indicated in bold.

*Adiponectin, levels in PDR patients above the highest standards; IGFBP6, NrCAM, TIMP-1, all levels above the highest standards; PlGF, all control levels were below LOD and set at LOD/√2.

**Fig 1:**
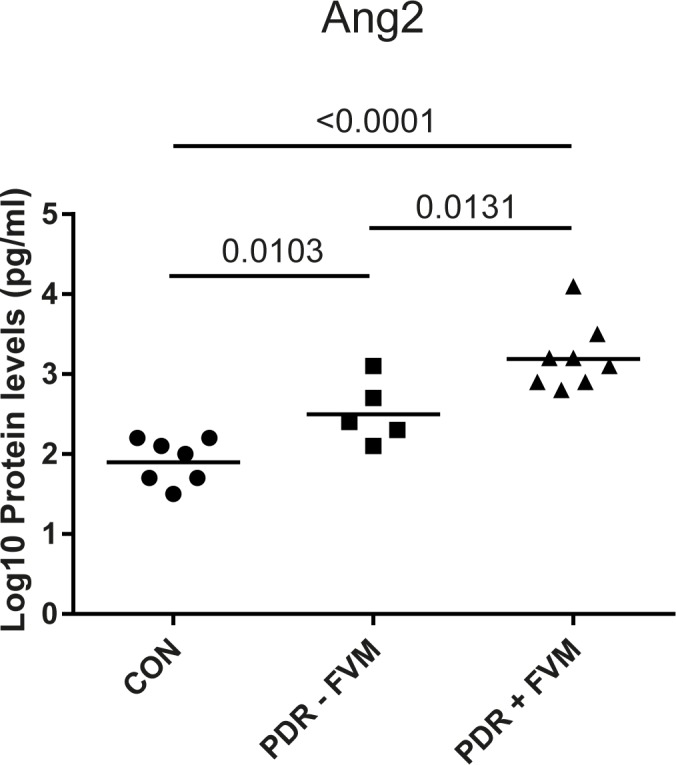
Ang2 protein is increased in vitreous of PDR patients with FVMs. Protein levels of Ang2, as detected by Quantibody arrays, were log10 transformed to obtain a normal distribution. Differences between groups were analyzed by a Student's t-test. CON, non-diabetic control patients; PDR–FVM, PDR patients without FVMs; PDR + FVM, PDR patients with FVMs. The lines represent the geometric means.

Next, we attempted to identify differences in protein concentrations between PDR patients with and without FVMs. Vitreous samples were divided into control, PDR patients without membranes and PDR patients with membranes. A significant upregulation of Ang2 in PDR+FVM patients compared to PDR-FVM patients was found (Kruskal-Wallis + Mann-Whitney U test) (Fig 1). Ang2 protein levels were log_10_ transformed to obtain a normal distribution. A similar trend for Ang2 was observed in the independent study group (S1 Fig). No statistically significant differences in concentrations of the other proteins were found in PDR patients with or without FVMs.

### Correlations between vitreous protein concentrations and clinical features of PDR

Correlations between vitreous protein concentrations in PDR patients and clinical features of fibrosis, neovascularization and vitreous hemorrhage were assessed using Spearman’s rank correlation coefficients. Moderate correlations were defined as coefficients between 0.40 and 0.59 and strong correlations between 0.60–0.79. PDGF-AA, Ang2 and PlGF showed a strong correlation with the degree of fibrosis (R^2^ = 0.638, 0.632 and 0.597, respectively), whereas IGF-II R showed a strongly negative correlation (R^2^ = -0.617). Additionally, IGFBP-6, GDF-15 and Ang1 were strongly correlated with neovascularization (R^2^ = 0.629, 0.623 and 0.599, respectively). The degree of vitreous hemorrhage was negatively correlated with NrCAM, NCAM-1 and DcR3 (R^2^ = 0.709, 0.669 and 0.563, respectively).

### Effects of bevacizumab on protein concentrations

**Fig 2:**
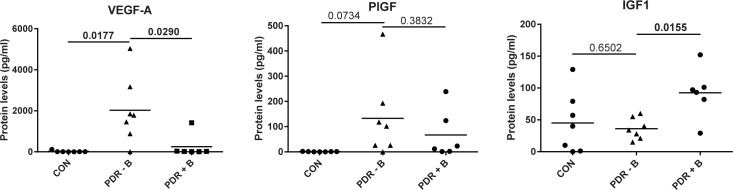
Effect of anti-VEGF therapy on protein levels. Samples were divided in three groups: CON, non-diabetic control patients; PDR—B, PDR patients that did not receive bevacizumab; PDR + B, PDR patients that received bevacizumab. Differences between groups were analyzed with an unpaired t test with Welch's correction. Lines represent mean values.

Six of the PDR patients had been treated by intravitreous injection with bevacizumab 3 days prior to surgery. We investigated whether the use of bevacizumab had effects on the concentrations of proteins other than VEGF. Whereas VEGF concentrations were almost completely downregulated by bevacizumab, concentrations of PlGF (a family member of VEGF) were not significantly affected (Fig 2). IGF1 concentrations, however, were significantly higher in bevacizumab-treated eyes (p = 0.0155). Significant effects of bevacizumab on other proteins were not observed.

### Correlations between proteins

**Fig 3:**
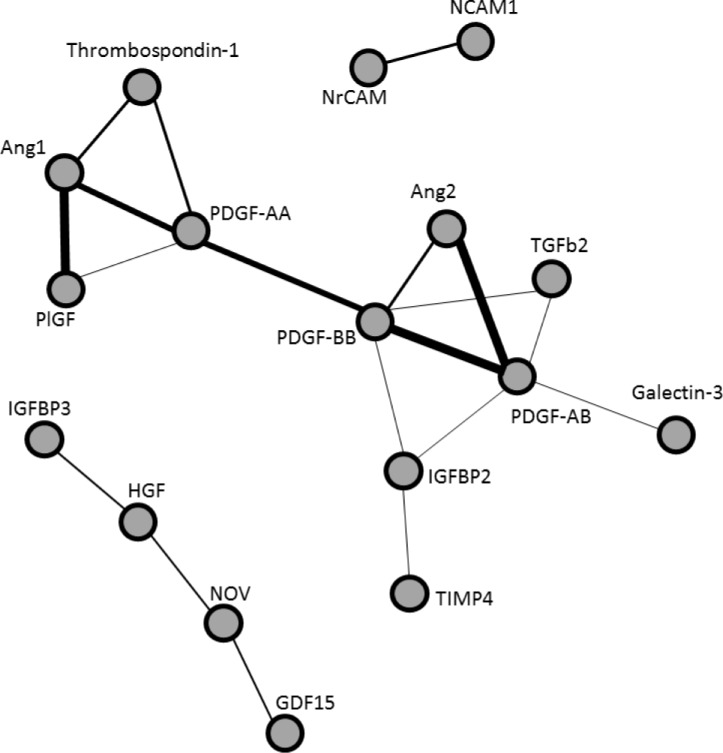
Coregulation of proteins. Pearson correlations between Quantibody protein profiles over all samples as calculated for all pairs of proteins. The proteins are presented in a correlation network in which the nodes represent proteins and the interconnecting lines represent the correlation between the proteins. The thickness of the lines represent the strength of the correlation. All correlations <0.7 and disconnected proteins were removed.

We investigated whether proteins were co-regulated and analyzed this by a pairwise comparison of proteins (Fig 3). Visual inspection revealed 3 groups of co-expressed proteins. The largest group contained the most interactions, showing co-expression of various proteins centered around a PDGF axis, consisting of the PDGF-AA, AB and BB subunits. In another group, co-regulation was found between neuronal cell adhesion molecule (NrCAM) and neural cell adhesion molecule (NCAM). Weaker associations were found in the third group between growth differentiation factor 15 (GDF15), nephroblastoma overexpressed (NOV), hepatocyte growth factor (HGF), and insulin-like growth factor binding protein 3 (IGFBP3).

### Gene expression in idiopathic and PDR-associated membranes

In order to identify possible cellular or tissue sources of the proteins in the vitreous of PDR patients, we determined gene expression in FVMs and compared these data to gene expression in blood cells from diabetic patients, control retina tissue, retinal pigment epithelial cells (RPE), human retinal endothelial cells (HRECs), human retinal pericytes (HRPCs) and human glial cells (Table 4). In total 11 idiopathic ERMs, and 10 PDR membranes were analyzed.

**Table 4:** Gene expression in membranes, retinal tissues and cells, and blood.

	n = 11	n = 10			Relative expression					
Gene	iERM	PDR	iERM	PDR*	Retina	RPE	Glial cells	HRECs	HRPCs	Blood
ADIPOQ	0	0	0	0	0	0	0	0	0	2
ANGPT2	7	9	30	135	150	0	4	22	0	13
BDNF	11	9	182	72	117	387	475	1337	550	1015
bNGF	8	6	7	32	13	394	134	30	210	687
GDF15	11	10	35	48	9	14	80	34	411	487
GDNF	11	10	12962	41091	0	0	156	177	0	512
HGF	10	8	136	359	268	3	0	72	857	153
ICAM1	11	9	90	224	780	545	24	279	75	1394
IGFBP1	7	7	26	47	0	1	4	721	7	56
IGFBP3	11	10	4179	2726	282	10095	317	3384	5234	257
NCAM1	10	8	810	392	1006	11	176	241	0	19
NGFR	10	8	1295	1933	1302	0	27	0	0	0
NOV	4	5	5	10	38	151	0	10	5	163
NRG1	10	5	36	12	47	99	649	318	33	17
NRG2	7	6	26	18	69	1	189	5	2	0
NRN1	11	9	288	392	3652	0	1628	169	0	979
NTF3	4	4	9	6	36	940	19	80	0.2	711
NTF4	0	0	0	0	0	0	101	3	5	773
PDGFA	0	2	0	4	0	0	11	0	0	17
PDGFB	0	0	0	0	0	0.5	3	14	0	29
PGF	9	7	242	47	71	0	10	15	0	5913
THBS1	11	10	2479	2011	2	610	88481	2749	7989	1132
TIMP1	11	10	6544	10088	11366	90200	40299	361760	59631	12807
VEGFA	11	10	4434	907	8879	18	8369	165	188	507

Left side (iERM, PDR): number of membranes in which gene expression is detected. Right side (Relative expression): abundance of gene expression in arbitrary units. iERM, idiopathic epiretinal membrane; PDR, epiretinal membrane from PDR patient; Retina, whole retina of non-diabetic donor eye; RPE, primary retinal pigment epithelial cells; Glial cells, U373 cell line; HRECs, human primary retinal endothelial cells; HRPCs, human primary retinal pericytes; Blood, pooled whole blood from patients with diabetic macular edema. Underlined values show significant difference in gene expression levels between PDR and iERM membranes (P < 0.05).

In accordance with protein concentrations found in the vitreous of PDR patients, 5 genes were found to be consistently expressed in all PDR membranes (*GDF15*, *IGFBP3*, *THBS1*, *TIMP1* and *VEGFA*). In addition, *BDNF* and *GDNF* were found to be expressed in all idiopathic and 9 of the 10 PDR membranes (Table 4), even though protein concentrations of these respective proteins in the vitreous were below the LOD (Table 3).

Increased vitreous protein concentrations may be due to either increased local production or due to leakage from the vasculature. Despite showing elevated vitreous concentrations, angiogenic growth factors such as adiponectin, *PDGFA* and *PDGFB* mRNAs were hardly, or not at all, found in membrane tissues. In other ocular tissues studied, adiponectin was only detected in blood cells and not in any of the ocular tissues including FVMs, whereas *PDGFA* was expressed in white blood cells and glial cells and *PDGFB* was expressed in white blood cells, RPE cells, glial cells and HRECs. Since PDGF concentrations in the vitreous of PDR patients were lower than the concentrations of PDGF reported in the plasma of both diabetic patients [34] and healthy volunteers [35], PDGFA is most likely derived from blood, whereas PDGFB is probably synthesized by either RPE, HREC or glial cells. *TIMP1*, which showed the highest vitreous protein concentrations, also showed the highest abundance in mRNA levels in all tissues and cells, with HRECs being the major source.

**Fig 4:**
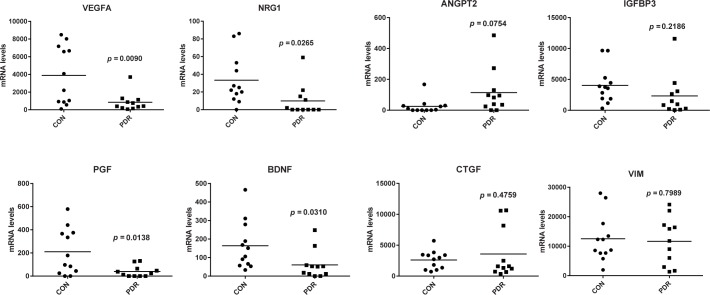
mRNA levels in idiopathic and PDR membranes. Transcript levels of genes were quantified by real-time quantitative PCR. Differences between groups were analyzed by a Student's t-test. Lines represent mean values.

When considering differences in mRNA expression levels between non-diabetic ERMs and PDR membranes, a few observations stand out: expression levels of *ANGPT2* were 4.6-fold higher in PDR membranes as compared to non-diabetic ERMs (Table 4; Fig 4), which is in agreement with the elevated vitreous protein concentrations in PDR patients (Table 3). Conversely, gene expression levels of *NRG1*, *PGF* and *VEGFA* were strikingly lower in PDR membranes when compared to controls (Table 4; Fig 4), whereas proteins concentrations in the vitreous of PDR patients were increased (Table 3). In addition, mRNA levels of connective tissue growth factor (*CTGF*), a marker of fibrosis [4], *IGFBP3* and vimentin (*VIM*), a highly abundant glial cell marker in ERMs, were comparable between non-diabetic ERMs and PDR membranes (Fig 4).

## Discussion

In the present study, vitreous samples were screened for 507 proteins, including various neural and glial growth factors, leading to the identification of 55 vitreous proteins elevated in PDR. A search in the Embase and Pubmed databases indicates that 8 of these proteins (BMP2, DcR3, GDF15, IGFBP4, NGFR, NOV, NRG1β1, UBB+1) were not previously reported to be associated with PDR.

A serious sight threatening complication of PDR is the formation of FVMs, which may result in retinal traction and retinal detachment. An initial step in the formation of these membranes may be neurite outgrowth from neurons and Müller cells, which not only secrete neurotrophic and pro-angiogenic growth factors, but may also serve as a template for the growth of other cell types and new blood vessels [4,18,19]. We identified Ang2 as being the most strongly correlated protein to the degree of fibrosis and the presence of FVMs in patients with PDR. Furthermore, the PDGFs seem to play a role in fibrosis and in neurite outgrowth as well, based on our observations in all membranes.

### Proteins related to neurite outgrowth

Many of the tested neurotrophins, such as NT3, NT4, bNGF, BDNF and GDNF were below the LOD in both control samples and PDR samples, suggesting that these proteins are not involved in retinal-vitreous neurite formation. Other studies confirmed the absence of bNGF and BDNF [36], but showed detectable levels of NT3 and NT4 [36] and GDNF [37], suggesting differences in sensitivity or specificity of the detection methods. While GDNF was found to be undetectable in the vitreous of PDR patients, we did find active mRNA transcription of *GDNF* in all PDR-associated membranes, as well as in the non-diabetic macular puckers. Further studies to clarify the possible role that GDNF may play in retinal neurite growth are warranted.

Other neurotrophic factors such as Neuregulin1/Heregulin1-b1 (NRG-1) exhibited a >3 fold upregulation in the vitreous of PDR patients, implicating their possible involvement in neurite outgrowth. Indeed, previous reports show that NRG1 can elicit neurite outgrowth in dorsal root ganglia explants [38] and developing rat retina [39]. Additionally, there may be increased sensitivity for neurotrophic factors as evidenced by the elevated concentrations of nerve growth factor receptor (NGFR) found in the vitreous of PDR patients. However, in the independent study group, the increased NRG1β1 protein levels were not confirmed, thus making the involvement of NRG1β1 in neurite outgrowth dubious.

Another possibility is that PDGF drives neurite growth, in addition to its angiogenic effects [40]. Indeed, PDGF has been shown to induce neuronal [41,42] and Müller cell proliferation *in vitro* [43], and outgrowth of neurites in primary rat brain cultures [44]. Additionally, transgenic mouse models overexpressing PDGFA have increased retinal glial cell proliferation [45], which can in turn drive neurite outgrowth via purinergic G protein-coupled receptor activation [46].

### A role for PDGFs in fibrosis

Although PDGF is known to be a strong angiogenic stimulus, we also found a strong correlation between PDGFAA concentrations and retinal fibrosis. In addition, PDGF was correlated with a number of other proteins known to be involved in fibrosis and neovascularization such as angiopoietin-1 (Ang1, ANGPT1) and -2 (Ang2, ANGPT2), PlGF and TGFβ [47]. This raises the possibility that PDGF may be one of the driving forces behind pathological angiogenesis and fibrosis in patients with PDR. Recent studies have shown that PDGF has a role in the production of FVMs in patients with a related condition, proliferative vitreoretinopathy (reviewed by Lei *et al*., 2010) [48]. In this condition, in contrast to PDR, fibrosis rather than a mixture of angiogenesis and fibrosis is the main pathological process.

### Angiopoietins

Ang1 and Ang2 are known to exert their biological effects by competitively binding to endothelial cell-specific tyrosine kinase with immunoglobulin and epidermal growth factor homology domains 2 (Tie-2) receptors [49]. The angiopoietin/Tie2 system is a context-dependent system with opposing effects. Binding of Ang1 leads to Tie2 phosphorylation and endothelial cell stabilization, but Ang2 binding leads to robust angiogenesis in the presence of VEGF [50]. A decreased ratio of Ang1 and Ang2 seems to be a critical switch for the development of vascular pathology, including blood-retinal barrier breakdown [51] and pericyte migration [52]. Here, we confirm reports that PDR is characterized by a disturbed balance of Ang1 and Ang2, since the concentrations of Ang2 were found to be increased almost 20-fold in PDR, whereas Ang1 concentrations only increased 3-fold.

In addition, Ang2 was found to be correlated to the degree of retinal fibrosis. *ANGPT2* gene expression was found in all PDR membranes and in the majority of non-diabetic ERMs. Furthermore, vitreous samples of PDR with FVMs had increased concentrations of Ang2 compared to PDR without FVMs. This could be accounted for by increased production of Ang2 from highly activated FVM endothelial cells [53]. It seems that, in addition to playing a role in active angiogenesis [50,54], Ang2 may well be involved in retinal fibrosis in PDR. This is supported by increased intravitreal Ang2 concentrations related to rhegmatogenous retinal detachment [55] and formation of FVMs in patients with retinopathy of prematurity [56]. Also, Ang2 was shown to be causative in the formation of liver fibrosis in rats [57] and cardiac fibrosis in db/db mice [58].

### Thrombospondin

We observed high protein concentrations of thrombospondin-1 in vitreous of PDR patients, 10-fold higher than in control vitreous samples. In conjunction, gene expression of *THBS1* was observed in all membranes in comparable quantities in non-diabetic ERMs and PDR membranes. Thrombospondin-1 is a matricellular glycoprotein that has anti-angiogenic properties and is involved in wound healing and fibrosis in the eye [59]. A possible mechanism is the activation of latent TGFβ, either directly [60], or indirectly by induction of MMP-2 and MMP-9 [61,62]. It is detected as an abundant protein on platelets and is secreted by endothelial cells, fibroblasts, smooth muscle cells, and many other cells of the retina, including glial cells (summarized by Masli *et al*., 2014 [59]). Indeed, we observed high transcript levels in these cell types, especially in glial cells, endothelial cells and pericytes. Thrombospondin-1 protein in vitreous has not been widely studied thus far. One study reports undetectable levels [63], whereas another study reports detectable levels by western blotting in human postmortem donor eyes and rat eyes [64]. In contrast to our results, vitreous samples from diabetic rats showed decreased thrombospondin-1 levels as compared to controls in the prior study. More research is needed to find out whether these differences are caused by technical or species-dependent factors. Considering the contribution to anti-angiogenesis, thrombospondin-1 may be upregulated in response to pro-angiogenic factors to counteract and balance the angiogenic switch in PDR. Interestingly, thrombospondin-1 and Ang2 were both clustered around the PDGF-axis in the correlation network analysis, which suggests that these proteins are co-regulated within our samples. This may mean that PDGFs, thrombospondin-1 and Ang2 work together in the formation of FVMs in PDR. More research is needed to clarify the relationship between these proteins.

### IGFBPs

Two insulin-like growth factor binding proteins, IGFBP-1 and -3, stood out with respect to their increased vitreous protein concentrations in PDR patients as compared to controls, with an increase of 10- and 19-fold respectively. Of these, *IGFBP3* mRNA was also abundantly expressed in non-diabetic ERMs and PDR membranes and several control tissues, with RPE cells showing the most abundant expression. Others have reported increased IGFBP1 [65] and IGFBP3 [66,67] levels in the vitreous of PDR patients in the same range. IGFBPs bind IGFs in the serum to protect them from degradation and increase their bioavailability [68]. IGFBP3 is described to have both pro-angiogenic and anti-angiogenic effects, probably dependent on the context and the presence of certain other growth factors. IGF1- and VEGF-induced proliferation and survival of human umbilical vein endothelial cells were both inhibited by IGFBP3 [69], whereas IGFBP3 supplementation to mouse Matrigel implants increased vascular ingrowth as compared to control animals [70]. In addition, IGFBP3 has been reported to be a protector of blood-retinal barrier breakdown and a stimulator of vasorelaxation by mediating NO levels [71]. Because of complex interactions with other serum proteins, the mechanism of IGFBP3 involvement in the pathophysiology of PDR may be difficult to unravel. On the other hand, IGFBP3 levels may serve as a useful biomarker of disease severity in PDR.

### Effect of bevacizumab

Because some of the patients received bevacizumab before surgery, we were able to analyze the effects of anti-VEGF treatment on protein concentrations. There have been concerns that inhibition of VEGF may upregulate other pro-angiogenic proteins, which could explain resistance to anti-VEGF therapy. One such protein is PlGF, a family member of VEGF, which has been reported to be iatrogenically upregulated by anti-VEGF therapy [72,73]. In the present study, we did not observe an upregulation on PlGF following bevacizumab treatment, whereas concentrations of IGF1 were significantly upregulated. These observations need to be carefully interpreted since the number of patients was rather low and need further investigations. IGF1 upregulation may be a reaction to the absence of VEGF, since IGF1 is known to induce VEGF synthesis [74]. It is worth noting that mRNA levels of VEGF were also significantly lower in the membranes of PDR patients as compared to controls. It is possible that this may due to the anti-VEGF treatment since all except two of the PDR patients received bevacizumab before surgery. It has previously been reported that anti-VEGF treatment may target endogenous VEGF levels, which is known to regulate its own transcription through VEGFR2 [75]. Besides VEGF, concentrations of 3 other protein were decreased in ERMs of PDR patients as compared to controls: PlGF, NRG1 and BDNF. Decreased PlGF may be explained by the transcriptional regulation by VEGF in microvascular endothelial cells [76], but the relation of VEGF with NRG1 and BDNF is less clear. In one study VEGF was found to work upstream from BDNF produced by endothelial cells in neurogenesis in songbirds [77] and in another study BDNF was found to promote the expression of NRG1 in neurons [78]. These observations warrant further investigation on larger patient groups.

### Limitations of the study

This study was limited by the small number of samples that we were able to obtain. Future studies using a larger patient population will enhance the statistical power of the correlations obtained in this study. Additionally, the use of a non-proliferative diabetic group of patients will allow more accurate assessment which growth factors are specific to PDR.

The cross-sectional acquisition of vitreous fluid only allowed protein measurements to be taken at a single time point in the course of disease. This makes inferences regarding causality difficult since the sequence of fluctuations in protein concentrations over time could not be obtained. Furthermore, we were unable to distinguish between proteins produced in the retina and proteins derived from the blood via a compromised blood-retina barrier or vitreous hemorrhage [79].

Our selection of proteins analyzed in the antibody arrays was limited by commercial availability. In this way, we regret that it was not possible to include measurements of CTGF, a protein previously reported to be integral in the relationship between retinal fibrosis and angiogenesis [4]. Further studies investigating the changes, origin, and sites of action of the proteins identified here will yield further insight into the pathophysiology of FVM formation in PDR.

## Conclusions

In conclusion, we found 8 novel proteins in the vitreous of PDR patients, as well as novel correlations between vitreous proteins and retinal fibrosis and angiogenesis. Ang2 was not only strongly related to the degree of fibrosis, but was also related to the occurrence of FVMs, suggesting that it may have a causative role in the formation of these membranes. In addition, the elevated concentrations of the neurotrophic factors NRG1 and PDGF in vitreous, and high gene expression levels of *GDNF* and *BDNF* in PDR membranes present potential proteins responsible for the retinal neurite growth that is displayed in all FVMs. The large amount of proteins that have been screened in our study may serve as a basis for more detailed analysis in larger study groups, eventually leading to a better understanding of molecular mechanisms of PDR pathology.

## Supporting information

S1 Table Significantly modulated human vitreous proteins. Proteins in vitreous detected by the RayBio® L‐Series 507 Biotin Label‐based Antibody Array that were significantly modulated in a subset of PDR patients (n = 7) relative to non‐diabetic controls (n = 5).
(PDF)

S2 Table Primer details. Gene nomenclature, Gen bank accession code, primer sequences, and size and predicted Tm of the amplified products.
(PDF)

S3 Table Protein concentrations in vitreous of control and PDR patients in independent study group. Protein concentrations were determined by Quantibody arrays in patients with macular hole (CON) and patients with proliferative diabetic retinopathy (PDR) that underwent vitreoretinal surgery.
(PDF)

S1 Fig Ang2 protein is increased in vitreous of PDR patients with FVMs. In an independent study group protein levels of Ang2, as detected by Quantibody arrays, were log10 transformed to obtain a normal distribution. Differences between groups were analyzed by a Student's t-test. CON, non-diabetic control patients; PDR–FVM, PDR patients without FVMs; PDR + FVM, PDR patients with FVMs. The lines represent the geometric means.
(PDF)

## References

[1] Gulkilik G, Taskapili M, Kocabora S, Muftuoglu G, Demirci G. Intravitreal bevacizumab for persistent macular edema with proliferative diabetic retinopathy. Int Ophthalmol. 2010; 30: 697–702. doi: 10.1007/s10792-010-9403-y 20936526 10.1007/s10792-010-9403-y

[2] Elman MJ, Ayala A, Bressler NM, Browning D, Flaxel CJ, Glassman AR, et al Intravitreal Ranibizumab for diabetic macular edema with prompt versus deferred laser treatment: 5-year randomized trial results. Ophthalmology. 2015; 122: 375–381. doi: 10.1016/j.ophtha.2014.08.047 25439614 10.1016/j.ophtha.2014.08.047 PMC4520307

[3] Bringmann A, Wiedemann P. Involvement of Müller glial cells in epiretinal membrane formation. Graefes Arch Clin Exp Ophthalmol. 2009; 247: 865–883. doi: 10.1007/s00417-009-1082-x 19415318 10.1007/s00417-009-1082-x

[4] Kuiper EJ, Van Nieuwenhoven FA, de Smet MD, van Meurs JC, Tanck MW, Oliver N, et al The angio-fibrotic switch of VEGF and CTGF in proliferative diabetic retinopathy. PLoS One. 2008; 3: e2675 doi: 10.1371/journal.pone.0002675 18628999 10.1371/journal.pone.0002675 PMC2443281

[5] Van Geest RJ, Lesnik-Oberstein SY, Tan HS, Mura M, Goldschmeding R, Van Noorden CJ, et al A shift in the balance of vascular endothelial growth factor and connective tissue growth factor by bevacizumab causes the angiofibrotic switch in proliferative diabetic retinopathy. Br J Ophthalmol. 2012; 96: 587–590. doi: 10.1136/bjophthalmol-2011-301005 22289291 10.1136/bjophthalmol-2011-301005 PMC3308470

[6] Li J-K, Wei F, Jin X-H, Dai Y-M, Cui H-S, Li Y-M. Changes in vitreous VEGF, bFGF and fibrosis in proliferative diabetic retinopathy after intravitreal bevacizumab. Int J Ophthalmol. 2015; 8: 1202–1206. doi: 10.3980/j.issn.2222-3959.2015.06.22 26682173 10.3980/j.issn.2222-3959.2015.06.22 PMC4651889

[7] Osaadon P, Fagan XJ, Lifshitz T, Levy J. A review of anti-VEGF agents for proliferative diabetic retinopathy. Eye (Lond). 2014; 28: 510–520. 24525867 10.1038/eye.2014.13 PMC4017101

[8] Neely KA, Gardner TW (1998). Ocular neovascularization: clarifying complex interactions. Am J Pathol. 1998; 153: 665–670. doi: 10.1016/S0002-9440(10)65607-6 9736014 10.1016/S0002-9440(10)65607-6 PMC1852998

[9] Frank RN. Diabetic Retinopathy. N Engl J Med. 2004; 350: 48–58. doi: 10.1056/NEJMra021678 14702427 10.1056/NEJMra021678

[10] Barber AJ, Gardner TW, Abcouwer SF. The significance of vascular and neural apoptosis to the pathology of diabetic retinopathy. Investig Ophthalmol Vis Sci. 2011; 52: 1156–1163. 21357409 10.1167/iovs.10-6293 PMC3053099

[11] Simo R, Hernandez C. Neurodegeneration is an early event in diabetic retinopathy: therapeutic implications. Br J Ophthalmol. 2012; 96: 1285–1290. doi: 10.1136/bjophthalmol-2012-302005 22887976 10.1136/bjophthalmol-2012-302005

[12] Lesnik Oberstein SY, Lewis GP, Dutra T, Fisher SK. Evidence that neurites in human epiretinal membranes express melanopsin, calretinin, rod opsin and neurofilament protein. Br J Ophthalmol. 2011; 95: 266–272. doi: 10.1136/bjo.2010.180679 20971788 10.1136/bjo.2010.180679

[13] Lesnik Oberstein SY, Lewis GP, Chapin EA, Fisher SK. Ganglion cell neurites in human idiopathic epiretinal membranes. Br J Ophthalmol. 2008; 92: 981–985. doi: 10.1136/bjo.2007.132332 18577651 10.1136/bjo.2007.132332

[14] Lewis GP, Betts KE, Sethi CS, Charteris DG, Lesnik-Oberstein SY, Avery RL, et al Identification of ganglion cell neurites in human subretinal and epiretinal membranes. Br J Ophthalmol. 2007; 91: 1234–1238. doi: 10.1136/bjo.2006.104612 17108012 10.1136/bjo.2006.104612 PMC1954915

[15] Sapieha P, Sirinyan M, Hamel D, Zaniolo K, Joyal JS, Cho JH, et al The succinate receptor GPR91 in neurons has a major role in retinal angiogenesis. Nat Med. 2008; 14: 1067–1076. doi: 10.1038/nm.1873 18836459 10.1038/nm.1873

[16] Dorrell MI, Aguilar E, Friedlander M. Retinal vascular development is mediated by endothelial filopodia, a preexisting astrocytic template and specific R-cadherin adhesion. Invest Ophthalmol Vis Sci. 2002; 43: 3500–3510. 12407162

[17] Siemerink MJ, Klaassen I, Van Noorden CJ, Schlingemann RO. Endothelial tip cells in ocular angiogenesis: potential target for anti-angiogenesis therapy. J Histochem Cytochem. 2013; 61:101–115. doi: 10.1369/0022155412467635 23092791 10.1369/0022155412467635 PMC3636692

[18] Snead DR, James S, Snead MP. Pathological changes in the vitreoretinal junction 1: epiretinal membrane formation. Eye (Lond). 2008; 22: 1310–1317. 18344963 10.1038/eye.2008.36

[19] Oberstein SY, Byun J, Herrera D, Chapin EA, Fisher SK, Lewis GP. Cell proliferation in human epiretinal membranes: characterization of cell types and correlation with disease condition and duration. Mol Vis. 2011; 17: 1794–1805. 21750605 PMC3133557

[20] Klaassen I, van Geest RJ, Kuiper EJ, van Noorden CJF, Schlingemann RO. The role of CTGF in diabetic retinopathy. Exp Eye Res 2015; 133: 37–48. doi: 10.1016/j.exer.2014.10.016 25819453 10.1016/j.exer.2014.10.016

[21] Suzuki Y, Nakazawa M, Suzuki K, Yamazaki H, Miyagawa Y. Expression profiles of cytokines and chemokines in vitreous fluid in diabetic retinopathy and central retinal vein occlusion. Jpn J Ophthalmol. 2011; 55: 256–263. doi: 10.1007/s10384-011-0004-8 21538000 10.1007/s10384-011-0004-8

[22] Boulton M, Gregor Z, McLeod D, et al Intravitreal growth factors in proliferative diabetic retinopathy: correlation with neovascular activity and glycaemic management. Br J Ophthalmol. 1997; 81: 228–233. 9135388 10.1136/bjo.81.3.228 PMC1722140

[23] Martinez-Zapata MJ, Martí-Carvajal AJ, Solà I, Pijoán JI, Buil-Calvo JA, Cordero JA, et al Anti-vascular endothelial growth factor for proliferative diabetic retinopathy. Cochrane Database Syst Rev. 2014; 11: CD008721. 10.1002/14651858.CD008721.pub2 PMC6995643 25418485

[24] Hinton DR, He S, Jin ML, Barron E, Ryan SJ. Novel growth factors involved in the pathogenesis of proliferative vitreoretinopathy. Eye (Lond). 2002; 16: 422–428. 12101449 10.1038/sj.eye.6700190

[25] Cui JZ, Chiu A, Maberley D, Ma P, Samad A, Matsubara JA. Stage specificity of novel growth factor expression during development of proliferative vitreoretinopathy. Eye (Lond). 2007; 21: 200–208. 16531976 10.1038/sj.eye.6702169

[26] Kita T, Clermont AC, Murugesan N, Zhou Q, Fujisawa K, Ishibashi T, et al Plasma Kallikrein-Kinin System as a VEGF-Independent Mediator of Diabetic Macular Edema. Diabetes. 2015; 64: 3588–3599. doi: 10.2337/db15-0317 25979073 10.2337/db15-0317 PMC4587649

[27] Smith JM, Steel DH. Anti-vascular endothelial growth factor for prevention of postoperative vitreous cavity haemorrhage after vitrectomy for proliferative diabetic retinopathy. Cochrane Database Syst Rev. 2015; 8: CD008214. 10.1002/14651858.CD008214.pub3 PMC6599827 26250103

[28] Ramos de Carvalho JE, Klaassen I, Vogels IM, Schipper-Krom S, van Noorden CJ, Reits E, et al Complement factor C3a alters proteasome function in human RPE cells and in an animal model of age-related RPE degeneration. Invest Ophthalmol Vis Sci. 2013; 54: 6489–6501. doi: 10.1167/iovs.13-12374 23982842 10.1167/iovs.13-12374

[29] Wisniewska-Kruk J, Hoeben KA, Vogels IM, Gaillard PJ, Van Noorden CJ, Schlingemann RO, etal A novel co-culture model of the blood-retinal barrier based on primary retinal endothelial cells, pericytes and astrocytes. Exp Eye Res. 2012; 96: 181–190. doi: 10.1016/j.exer.2011.12.003 22200486 10.1016/j.exer.2011.12.003

[30] Fickweiler W, Klaassen I, Vogels IM, Hooymans JM, Wolffenbuttel BH, Los LI, et al Association of Circulating Markers With Outcome Parameters in the Bevacizumab and Ranibizumab in Diabetic Macular Edema Trial. Invest Ophthalmol Vis Sci. 2016; 57: 6234–6241. doi: 10.1167/iovs.16-20157 27842163 10.1167/iovs.16-20157

[31] Klaassen I, Hughes JM, Vogels IMC, Schalkwijk CG, Van Noorden CJF, Schlingemann RO. Altered expression of genes related to blood-retina barrier disruption in streptozotocin-induced diabetes. Exp Eye Res. 2009; 89: 4–15. doi: 10.1016/j.exer.2009.01.006 19284967 10.1016/j.exer.2009.01.006

[32] Hellemans J, Vandesompele J. Selection of reliable reference genes for RT-qPCR analysis. Methods Mol Biol. 2014; 1160: 19–26. doi: 10.1007/978-1-4939-0733-5_3 24740218 10.1007/978-1-4939-0733-5_3

[33] Ogden TL. Handling results below the level of detection. Ann Occup Hyg. 2010;54: 255–256. doi: 10.1093/annhyg/mep099 20067938 10.1093/annhyg/mep099

[34] Harrison AA, Dunbar PR, Neale TJ. Immunoassay of platelet-derived growth factor in the blood of patients with diabetes mellitus. Diabetologia. 1994; 37: 1142–1146. 7867886 10.1007/BF00418378

[35] Biancotto A, Feng X, Langweiler M, Young NS, Philip McCoy J. Effect of anticoagulants on multiplexed measurement of cytokine/chemokines in healthy subjects. Cytokine. 2012; 60: 438–446. doi: 10.1016/j.cyto.2012.05.019 22705152 10.1016/j.cyto.2012.05.019 PMC3449030

[36] Abu El-Asrar AM, Mohammad G, De Hertogh G, Nawaz MI, Van Den Eynde K, Siddiquei MM, et al Neurotrophins and neurotrophin receptors in proliferative diabetic retinopathy. PLoS One. 2013; 8: e65472 doi: 10.1371/journal.pone.0065472 23762379 10.1371/journal.pone.0065472 PMC3676317

[37] Nishikiori N, Mitamura Y, Tashimo A, Nakamura Y, Harada T, Osanai M, et al Glial cell line-derived neurotrophic factor in the vitreous of patients with proliferative diabetic retinopathy. Diabetes Care. 2005; 28: 2588. 10.2337/diacare.28.10.2588 16186306

[38] Liu Z, Gao W, Wang Y, Zhang W, Liu H, Li Z. Neuregulin-1β regulates outgrowth of neurites and migration of neurofilament 200 neurons from dorsal root ganglial explants in vitro. Peptides. 2011; 32: 1244–1248. doi: 10.1016/j.peptides.2011.04.005 21515322 10.1016/j.peptides.2011.04.005

[39] Bermingham-McDonogh O, McCabe KL, Reh TA. Effects of GGF/neuregulins on neuronal survival and neurite outgrowth correlate with erbB2/neu expression in developing rat retina. Development. 1996; 122: 1427–1438. 8625831 10.1242/dev.122.5.1427

[40] Battegay EJ, Rupp J, Iruela-Arispe L, Sage EH, Pech M. PDGF-BB modulates endothelial proliferation and angiogenesis in vitro via PDGF beta-receptors. J Cell Biol. 1994; 125: 917–928. 7514607 10.1083/jcb.125.4.917 PMC2120083

[41] Park N, Yoo JC, Ryu J, Hong S-G, Hwang EM, Park J-Y. Copine1 enhances neuronal differentiation of the hippocampal progenitor HiB5 cells. Mol Cells. 2012; 34: 549–554. doi: 10.1007/s10059-012-0235-7 23263657 10.1007/s10059-012-0235-7 PMC3887833

[42] Funa K, Sasahara M. The roles of PDGF in development and during neurogenesis in the normal and diseased nervous system. J Neuroimmune Pharmacol. 2014; 9: 168–181. doi: 10.1007/s11481-013-9479-z 23771592 10.1007/s11481-013-9479-z PMC3955130

[43] Moon SW, Chung EJ, Jung S-A, Lee JH. PDGF stimulation of Müller cell proliferation: Contributions of c-JNK and the PI3K/Akt pathway. Biochem Biophys Res Commun. 2009; 388: 167–171. doi: 10.1016/j.bbrc.2009.07.144 19653997 10.1016/j.bbrc.2009.07.144

[44] Smits A, Kato M, Westermark B, Nistér M, Heldin CH, Funa K. Neurotrophic activity of platelet-derived growth factor (PDGF): Rat neuronal cells possess functional PDGF beta-type receptors and respond to PDGF. Proc Natl Acad Sci U S A. 1991; 88: 8159–8163. 1654560 10.1073/pnas.88.18.8159 PMC52466

[45] Mori K, Gehlbach P, Ando A, Dyer G, Lipinsky E, Chaudhry AG, et al Retina-specific expression of PDGF-B versus PDGF-A: Vascular versus nonvascular proliferative retinopathy. Investig Ophthalmol Vis Sci. 2002; 43: 2001–2006. 12037011

[46] Taguchi M, Shinozaki Y, Kashiwagi K, Shigetomi E, Robaye B, Koizumi S. Müller cell-mediated neurite outgrowth of the retinal ganglion cells via P2Y6 receptor signals. J Neurochem. 2016; 136: 741–751. 10.1111/jnc.13427 26560804

[47] Schlingemann RO. Role of growth factors and the wound healing response in age-related macular degeneration. Graefes Arch Clin Exp Ophthalmol. 2004; 242: 91–101. doi: 10.1007/s00417-003-0828-0 14685874 10.1007/s00417-003-0828-0

[48] Lei H, Rheaume MA, Kazlauskas A. Recent developments in our understanding of how platelet-derived growth factor (PDGF) and its receptors contribute to proliferative vitreoretinopathy. Exp Eye Res. 2010; 90: 376–381. doi: 10.1016/j.exer.2009.11.003 19931527 10.1016/j.exer.2009.11.003 PMC2824005

[49] Nambu H, Nambu R, Oshima Y, et al Angiopoietin 1 inhibits ocular neovascularization and breakdown of the blood-retinal barrier. Gene Ther. 2004; 11: 865–873. doi: 10.1038/sj.gt.3302230 15042118 10.1038/sj.gt.3302230

[50] Watanabe D, Suzuma K, Suzuma I, Ohashi H, Ojima T, Kurimoto M, et al Vitreous levels of angiopoietin 2 and vascular endothelial growth factor in patients with proliferative diabetic retinopathy. Am J Ophthalmol. 2005; 139: 476–481. doi: 10.1016/j.ajo.2004.10.004 15767056 10.1016/j.ajo.2004.10.004

[51] Lobov IB, Brooks PC, Lang RA. Angiopoietin-2 displays VEGF-dependent modulation of capillary structure and endothelial cell survival in vivo. Proc Natl Acad Sci U S A. 2002; 99: 11205–11210. doi: 10.1073/pnas.172161899 12163646 10.1073/pnas.172161899 PMC123234

[52] Pfister F, Wang Y, Schreiter K, vom Hagen F, Altvater K, Hoffmann S, et al Retinal overexpression of angiopoietin-2 mimics diabetic retinopathy and enhances vascular damages in hyperglycemia. Acta Diabetol. 2010; 47: 59–64. doi: 10.1007/s00592-009-0099-2 19238311 10.1007/s00592-009-0099-2

[53] Abu El-Asrar AM, Missotten L, Geboes K. Expression of hypoxia-inducible factor-1alpha and the protein products of its target genes in diabetic fibrovascular epiretinal membranes. Br J Ophthalmol. 2007; 91: 822–826. doi: 10.1136/bjo.2006.109876 17229797 10.1136/bjo.2006.109876 PMC1955571

[54] Patel JI, Hykin PG, Gregor ZJ, Boulton M, Cree IA. Angiopoietin concentrations in diabetic retinopathy. Br J Ophthalmol. 2005; 89: 480–483. doi: 10.1136/bjo.2004.049940 15774928 10.1136/bjo.2004.049940 PMC1772595

[55] Loukovaara S, Lehti K, Robciuc A, Pessi T, Holopainen JM, Koli K, et al Increased intravitreal angiopoietin-2 levels associated with rhegmatogenous retinal detachment. Graefes Arch Clin Exp Ophthalmol. 2014; 252: 881–888. doi: 10.1007/s00417-013-2508-z 24218041 10.1007/s00417-013-2508-z

[56] Umeda N, Ozaki H, Hayashi H, Miyajima-Uchida H, Oshima K. Colocalization of Tie2, angiopoietin 2 and vascular endothelial growth factor in fibrovascular membrane from patients with retinopathy of prematurity. Ophthalmic Res. 2003; 35: 217–223. 12815197 10.1159/000071173

[57] Pauta M, Ribera J, Melgar-Lesmes P, Casals G, Rodríguez-Vita J, Reichenbach V, et al Overexpression of angiopoietin-2 in rats and patients with liver fibrosis. Therapeutic consequences of its inhibition. Liver Int. 2015; 35: 1383–1392. doi: 10.1111/liv.12505 24612347 10.1111/liv.12505

[58] Chen JX, Zeng H, Reese J, Aschner JL, Meyrick B. Overexpression of angiopoietin-2 impairs myocardial angiogenesis and exacerbates cardiac fibrosis in the diabetic db/db mouse model. Am J Physiol Heart Circ Physiol. 2012; 302: H1003–H1012. doi: 10.1152/ajpheart.00866.2011 22180648 10.1152/ajpheart.00866.2011 PMC3322731

[59] Masli S, Sheibani N, Cursiefen C, Zieske J. Matricellular protein thrombospondins: influence on ocular angiogenesis, wound healing and immuneregulation. Curr Eye Res. 2014; 39: 759–774. doi: 10.3109/02713683.2013.877936 24559320 10.3109/02713683.2013.877936 PMC4278647

[60] Schultz-Cherry S, Chen H, Mosher DF, Misenheimer TM, Krutzsch HC, Roberts DD, et al Regulation of transforming growth factor-beta activation by discrete sequences of thrombospondin 1. J Biol Chem. 1995; 270: 7304–7310. 7706271 10.1074/jbc.270.13.7304

[61] Lee T, Esemuede N, Sumpio BE, Gahtan V. Thrombospondin-1 induces matrix metalloproteinase-2 activation in vascular smooth muscle cells. J Vasc Surg. 2003; 38: 147–154. 12844104 10.1016/s0741-5214(02)75468-2

[62] Qian X, Wang TN, Rothman VL, Nicosia RF, Tuszynski GP. Thrombospondin-1 modulates angiogenesis in vitro by up-regulation of matrix metalloproteinase-9 in endothelial cells. Exp Cell Res. 1997; 235: 403–412. doi: 10.1006/excr.1997.3681 9299165 10.1006/excr.1997.3681

[63] Abu El-Asrar AM, Nawaz MI, Kangave D, Siddiquei MM, Ola MS, Opdenakker G. Angiogenesis regulatory factors in the vitreous from patients with proliferative diabetic retinopathy. Acta Diabetol. 2013; 50: 545–551. doi: 10.1007/s00592-011-0330-9 21947384 10.1007/s00592-011-0330-9

[64] Sheibani N, Sorenson CM, Cornelius LA, Frazier WA. Thrombospondin-1, a natural inhibitor of angiogenesis, is present in vitreous and aqueous humor and is modulated by hyperglycemia. Biochem Biophys Res Commun. 2000; 267: 257–261. doi: 10.1006/bbrc.1999.1903 10623607 10.1006/bbrc.1999.1903

[65] Hopkins KD, Brart DO, Russell-Jones DL, Chignell AH, Sönksen PH. Insulin-like growth factor binding protein-1 levels in diabetic proliferative retinopathy. Horm Metab Res. 1993; 25: 331–332. doi: 10.1055/s-2007-1002114 7688338 10.1055/s-2007-1002114

[66] Burgos R, Mateo C, Cantón A, Hernández C, Mesa J, Simó R. Vitreous levels of IGF-I, IGF binding protein 1, and IGF binding protein 3 in proliferative diabetic retinopathy: a case-control study. Diabetes Care. 2000; 23: 80–83. 10857973 10.2337/diacare.23.1.80

[67] Pfeiffer A, Spranger J, Meyer-Schwickerath R, Schatz H. Growth factor alterations in advanced diabetic retinopathy: a possible role of blood retina barrier breakdown. Diabetes. 1997; 46 Suppl 2: S26–S30. 9285495 10.2337/diab.46.2.s26

[68] Bach LA. Endothelial cells and the IGF system. J Mol Endocrinol. 2015; 54: R1–R13. doi: 10.1530/JME-14-0215 25351818 10.1530/JME-14-0215

[69] Franklin SL, Ferry RJ Jr, Cohen P. Rapid insulin-like growth factor (IGF)-independent effects of IGF binding protein-3 on endothelial cell survival. J Clin Endocrinol Metab. 2003; 88: 900–907. doi: 10.1210/jc.2002-020472 12574231 10.1210/jc.2002-020472 PMC3314536

[70] Granata R, Trovato L, Lupia E, Sala G, Settanni F, Camussi G, et al Insulin-like growth factor binding protein-3 induces angiogenesis through IGF-I- and SphK1-dependent mechanisms. J Thromb Haemost. 2007; 5: 835–845. doi: 10.1111/j.1538-7836.2007.02431.x 17388800 10.1111/j.1538-7836.2007.02431.x

[71] Jarajapu YP, Cai J, Yan Y, Li Calzi S, Kielczewski JL, Hu P, et al Protection of blood retinal barrier and systemic vasculature by insulin-like growth factor binding protein-3. PLoS One. 2012; 7: e39398 doi: 10.1371/journal.pone.0039398 22792172 10.1371/journal.pone.0039398 PMC3391198

[72] Huang H, He J, Johnson D, Wei Y, Liu Y, Wang S, et al Deletion of placental growth factor prevents diabetic retinopathy and is associated with Akt activation and HIF1α-VEGF pathway inhibition. Diabetes.2015; 64: 200–212. doi: 10.2337/db14-0016 25187372 10.2337/db14-0016 PMC4274802

[73] Zhou AY, Bai YJ, Zhao M, Yu WZ, Huang LZ, Li XX. Placental growth factor expression is reversed by antivascular endothelial growth factor therapy under hypoxic conditions. World J Pediatr. 2014; 10: 262–270. doi: 10.1007/s12519-014-0502-0 25124978 10.1007/s12519-014-0502-0

[74] Warren RS, Yuan H, Matli MR, Ferrara N, Donner DB. Induction of vascular endothelial growth factor by insulin-like growth factor 1 in colorectal carcinoma. J Biol Chem. 1996; 271: 29483–29488. 8910616 10.1074/jbc.271.46.29483

[75] Guangqi E, Cao Y, Bhattacharya S, Dutta S, Wang E, Mukhopadhyay D. Endogenous vascular endothelial growth factor-A (VEGF-A) maintains endothelial cell homeostasis by regulating VEGF receptor-2 transcription. J Biol Chem. 2012; 287: 3029–3041. doi: 10.1074/jbc.M111.293985 22167188 10.1074/jbc.M111.293985 PMC3270960

[76] Zhao B, Cai J, Boulton M. Expression of placenta growth factor is regulated by both VEGF and hyperglycaemia via VEGFR-2. Microvasc Res. 2004; 68: 239–246. doi: 10.1016/j.mvr.2004.07.004 15501243 10.1016/j.mvr.2004.07.004

[77] Louissaint A Jr, Rao S, Leventhal C, Goldman SA. Coordinated interaction of neurogenesis and angiogenesis in the adult songbird brain. Neuron. 2002; 34: 945–960. 12086642 10.1016/s0896-6273(02)00722-5

[78] Xiao J, Wong AW, Willingham MM, Kaasinen SK, Hendry IA, Howitt J, et al BDNF exerts contrasting effects on peripheral myelination of NGF-dependent and BDNF-dependent DRG neurons. J Neurosci. 2009; 29: 4016–4022. doi: 10.1523/JNEUROSCI.3811-08.2009 19339597 10.1523/JNEUROSCI.3811-08.2009 PMC6665359

[79] Simó-Servat O, Hernández C, Simó R. Usefulness of the vitreous fluid analysis in the translational research of diabetic retinopathy. Mediators Inflamm. 2012; 2012: 872978 doi: 10.1155/2012/872978 23028204 10.1155/2012/872978 PMC3457631

