# Supplementary material for: Identification of proteins associated with clinical and pathological features of proliferative diabetic retinopathy in vitreous and fibrovascular membranes
Source: PLoS One. 2017 Nov 2;12(11):e0187304. doi: 10.1371/journal.pone.0187304 (PMC5667868; doi:10.1371/journal.pone.0187304)
Supplement: S1 Table — Proteins in vitreous detected by the RayBio® L‐Series 507 Biotin Label‐based Antibody Array that were significantly modulated in a subset of PDR patients (n = 7) relative to non‐diabetic controls (n = 5). (PDF) [file pone.0187304.s001.pdf]

S1 Table. Significantly modulated human vitreous proteins. Proteins in vitreous detected by the RayBio® L-Series 507 Biotin Label-based Antibody Array that were significantly modulated in a subset of PDR patients (n = 7) relative to non-diabetic controls (n = 5).

**> 10-fold higher**					
**Name**	**PDR:Control**	**P-value**	**Name**	**PDR:Control**	**P-value**
XEDAR	2283.8	0.0244	IL-22 R	86.3	0.0055
IL-5 R alpha	1756.8	0.0074	NAP-2	62.3	0.0480
PD-ECGF	1318.9	0.0031	ICAM-1	53.2	0.0190
NT-4	447.8	0.0153	Thrombopoietin (TPO)	32.9	0.0059
Cerberus 1	421.9	0.0404	CRIM 1	32.4	0.0346
Ubiquitin+1	339.0	0.0018	IL-15 R alpha	22.3	0.0027
Orexin B	203.2	0.0055	IL-4	20.0	0.0090
Tarc	198.4	0.0196	Growth Hormone R (GHR)	18.4	0.0301
PlGF	151.9	0.0501	Growth Hormone (GH)	12.4	0.0065
IL-23 R	134.0	0.0148	CD40 Ligand / TNFSF5 /CD154	10.6	0.0060
TIMP-4	131.1	0.0097	CCR6	10.5	0.0122
IGFBP-3	108.1	0.0021	Insulin	10.4	0.0429
IL-2 R alpha	102.5	0.0107			
**2- to 10-fold higher**					
**Name**	**PDR:Control**	**P-value**	**Name**	**PDR:Control**	**P-value**
TGF-beta RI / ALK-5	10.0	0.0090	GFR alpha-3	3.8	0.0257
E-Selectin	8.7	0.0382	Adiponectin / Acrp30	3.8	0.0276
PDGF-AA	7.9	0.0089	CCL28 / VIC	3.6	0.0456
IFN-gamma R1	7.8	0.0059	Angiopoietin-1	3.6	0.0254
IL-17B R	7.2	0.0077	ICAM-3 (CD50)	3.5	0.0313
DcR3 / TNFRSF6B	6.8	0.0318	SIGIRR	3.5	0.0010
TGF-beta 5	6.6	0.0108	BMP-2	3.4	0.0345
IGFBP-2	6.4	0.0053	GREMLIN	3.2	0.0227
Thrombospondin (TSP)	5.8	0.0136	BMP-5	3.2	0.0191
NRG2	5.5	0.0239	Activin RIA / ALK-2	3.0	0.0329
TGF-beta 1	5.0	0.0430	Angiopoietin-2	2.7	0.0009
GDF-15	4.5	0.0011	Osteoprotegerin / TNFRSF11B	2.5	0.0404
IGFBP-1	4.3	0.0013	Neuritin	2.2	0.0154
Insulin R	4.3	0.0351	MFG-E8	2.1	0.0270
