# Supplementary material for: Identification of proteins associated with clinical and pathological features of proliferative diabetic retinopathy in vitreous and fibrovascular membranes
Source: PLoS One. 2017 Nov 2;12(11):e0187304. doi: 10.1371/journal.pone.0187304 (PMC5667868; doi:10.1371/journal.pone.0187304)
Supplement: S2 Table — Gene nomenclature, Gen bank accession code, primer sequences, and size and predicted Tm of the amplified products. (PDF) [file pone.0187304.s002.pdf]

Table S2. Primer details. Gene nomenclature, Gen bank accession code, primer sequences, and size (bp) and predicted Tm of the amplified products.

Unigene symbol	Description	GenBank	Forward primer	Reverse primer	bp	Tm
ADIPOQ	Adiponectin	NM_004797	GAAAGGAGATCCAGGTCTTATTGGTC	AAAGCCTCGGGGACCTTCAG	82	81
ANGPT2	Angiopoietin 2	NM_001147	GCAAAATAAGCAGCATCAGCCAAC	GCATCAAACCACCAGCCTCCT	115	76
BDNF	Brain-derived Neurotrophic Factor	NM_001143805	CCCAGTTCCACCAGGTGAGAA	CCTCGGATGTTTGCTTCTTTCAT	109	78
CTGF	Connective tissue growth factor	NM_001901	ACCAATGACAACGCCTCCTGC	CGGATGCACTTTTTGCCCTTCTTA	110	82
GDF15	Growth Differentiation Factor 15	NM_004864	ACGCTACGAGGACCTGCTAACC	CAGCCGCACTTCTGGCGTGAGTAT	112	85
GDNF	Glial Cell Line-derived Neurotrophic Factor	NM_000514	GCAGTGACTCAAATATGCCAGAGG	CCTGCCGATTCCGCTCTCTT	141	78
HGF	Hepatocyte Growth Factor	NM_000601	GGACCATGTGAGGGGGATTATG	CCACGACCAGGAACAATGACA	89	78
ICAM1	Intercellular Adhesion Molecule 1	NM_000201	GACAGGGCATTGTCCTCAGTCAGA	CAGATCAGATGCGTGGCCTAGTGT	87	79
IGFBP1	Insulin-like Growth Factor-Binding Protein 1	NM_000596	TGGAAGGAGCCCTGCCGAATA	GGATGTCTCACACTGTCTGCTGTG	147	79
IGFBP3	Insulin-like Growth Factor-Binding Protein 3	NM_000598	AAGACAGCCAGCGCTACAAAG	TACGGCAGGGACCATATTCTG	103	80
NCAM1	Neural Cell Adhesion Molecule 1	NM_000615	AAGACGCAGCCAGTCCAAGG	TGGTTTCCACTCGGAGGAGAGC	165	84
NGF	Nerve Growth Factor (beta-polypeptide)	NM_002506	CACTGAGGTGCATAGCGTAATGTC	CTGAGTGTGGTTCCGCCTGTAT	86	78
NGFR	Nerve growth factor receptor	NM_002507	GCCTACATAGCCTTCAAGAGGTGGA	TGCTGGTCATGCAGGCTCTG	152	84
NOV	Nephroblastoma Overexpressed	NM_002514	CAAGAGCCAGAGCAGCCAACAGAT	TGGTATTGTGGGGAGTGCAGCAG	169	83
NRG1	Neuregulin 1	NM_013956	CGTGGAATCAAACGAGATCATCA	GCTTGTCCCAGTGGTGGATGT	122	78
NRG2	Neuregulin 2	NM_004883	CCACGGAACAGCCCTTAGTCTTTA	CCGTCTGGCTCTTCATCTTCTTC	141	83
NTF3	Neurotrophin 3	NM_002527	GTCCGAGCACTGACTTCAGAGAA	AAGGCACACACACAGGACGTGTCTA	80	79
NTF4	Neurotrophin 4	NM_006179	TCGAATTGACACTGCCTGCGTCT	CCAAACTGGGGTCCTTAGATCAGC	136	84
PDGFA	Platelet Derived Growth Factor Subunit A	NM_002607	AAAGCAGCCAACCAGATGTGAGGT	CGGACACAGTTTTTCACGGAGGA	151	81
PDGFB	Platelet Derived Growth Factor Subunit B	NM_002608	TGCTGCTACCTGCGTCTGGTCA	CAGGCGTTGGAGATCATCAAAGGA	108	83
PlGF1	Human Placenta Growth Factor 1	NM_002632	TCACCATGCAGCTCCTAAAGATC	ACAGCATCGCCGCACCTTTC	133	85
THBS1	Thrombospondin 1	NM_003246	GCTGCACTGAGTGTCACTGTCAGAA	GCGAGGACAGCATTCTCCATCA	110	81
TIMP1	Tissue Inhibitor of Metalloproteinase 1	NM_003254	ACTTCCACAGGTCCCACAACCG	AGGGAAACACTGTGCATTCCTCAC	180	84
VIM	Vimentin	NM_003380	CAGATTCAGGAACAGCATGTCCAAA	AGGTCAGCAAACTTGGATTTGTACCA	152	81
