# Supplementary material for: Identification of proteins associated with clinical and pathological features of proliferative diabetic retinopathy in vitreous and fibrovascular membranes
Source: PLoS One. 2017 Nov 2;12(11):e0187304. doi: 10.1371/journal.pone.0187304 (PMC5667868; doi:10.1371/journal.pone.0187304)
Supplement: S3 Table — Protein concentrations were determined by Quantibody arrays in patients with macular hole (CON) and patients with proliferative diabetic retinopathy (PDR) that underwent vitreoretinal surgery. (PDF) [file pone.0187304.s003.pdf]

S3 Table. **Protein concentrations in vitreous of control and PDR patients in independent study group.**
Protein concentrations were determined by Quantibody arrays in patients with macular hole (CON) and patients with proliferative diabetic retinopathy (PDR) that underwent vitreoretinal surgery.

	CON (n=20)		PDR (n=32)		Fold	
	**mean**	**SD**	**mean**	**SD**	**Change**	**P-value**
ANG-2	697.8	915.2	16229.6	25984.5	**23.26**	**0.010**
NRG1-b1	2.7	0.6	5.8	13.5	2.12	0.315
PDGF-AA	371.8	252.0	1540.0	1816.7	**4.14**	**0.006**
PIGF	10.8	5.4	801.2	1280.1	**74.15**	**0.008**
VEGFA	117.3	96.4	23511.1	26858.9	**200.46**	**<0.001**

Unpaired t-test with Welch's correction was used to assess statistical differences between PDR and control patients. Fold changes higher than 3-fold and significant differences ($P < 0.05$) are indicated in bold.
