# Supplementary material for: Identification of proteins associated with clinical and pathological features of proliferative diabetic retinopathy in vitreous and fibrovascular membranes
Source: PLoS One. 2017 Nov 2;12(11):e0187304. doi: 10.1371/journal.pone.0187304 (PMC5667868; doi:10.1371/journal.pone.0187304)
Supplement: S1 Fig — In an independent study group protein levels of Ang2, as detected by Quantibody arrays, were log10 transformed to obtain a normal distribution. Differences between groups were analyzed by a Student's t-test. CON, non-diabetic control patients; PDR–FVM, PDR patients without FVMs; PDR + FVM, PDR patients with FVMs. The lines represent the geometric means. (PDF) [file pone.0187304.s004.pdf]

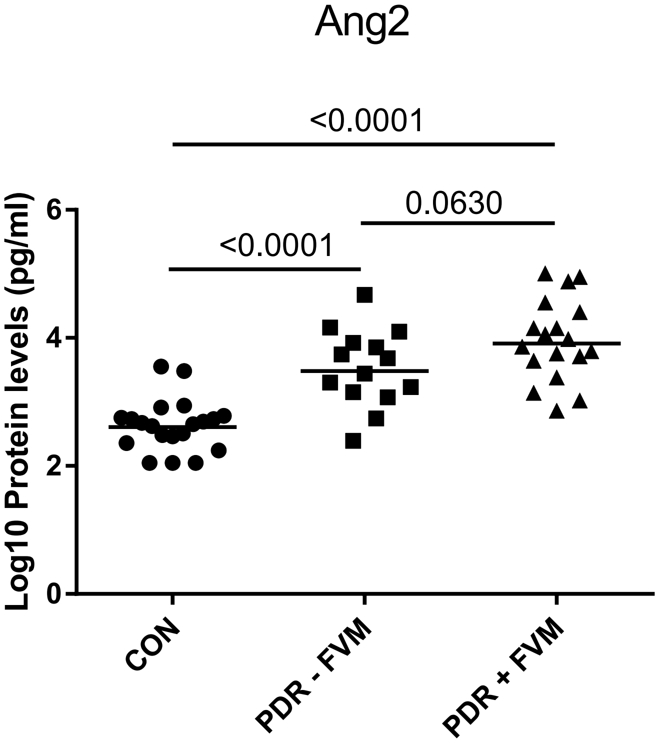
Ang2
<0.0001
0.0630
<0.0001
Log10 Protein levels (pg/ml)
6
4
2
0
CON
PDR - FVM
PDR + FVM
